# Enhancement of the Adhesive Strength between Ag Films and Mo Substrate by Ag Implanted via Ion Beam-Assisted Deposition

**DOI:** 10.3390/ma11050762

**Published:** 2018-05-09

**Authors:** Jiajun Zhu, Yuhao Hu, Meng Xu, Wulin Yang, Licai Fu, Deyi Li, Lingping Zhou

**Affiliations:** 1 College of Materials Science and Engineering, Hunan University, Changsha 410082, China; hyh0816@hnu.edu.cn (Y.H.); xumeng@hnu.edu.cn (M.X.); hnuywl@hnu.edu.cn (W.Y.); lfu@hnu.edu.cn (L.F.); lideyi@hnu.edu.cn (D.L.); 2 Hunan Province Key Laboratory for Spray Deposition Technology and Application, Hunan University, Changsha 410082, China

**Keywords:** Ag films, immiscible Ag-Mo system, adhesive strength, IBAD

## Abstract

Silver-coated molybdenum is an optimum material selection to replace pure silver as solar cell interconnector. However, the low adhesive strength between Ag films and Mo substrate hinders the application of the interconnector, because it is difficult to form metallurgical bonding or compound in the film/substrate interface using conventional deposition. In order to improve the adhesion, some Ag particles were implanted into the surface of Mo substrate by ion beam-assisted deposition (IBAD) before the Ag films were deposited by magnetron sputtering deposition (MD). The objective of this work was to investigate the effect of different assisted ion beam energy on the film/substrate adhesive properties. In addition, the fundamental adhesion mechanism was illustrated. The results revealed that the adhesion between Ag films and Mo substrate could be greatly enhanced by IBAD. With the increase of the assisting ion beam energy, the adhesive strength first increased and then decreased, with the optimum adhesion being able to rise to 25.29 MPa when the energy of the assisting ion beam was 30 keV. It could be inferred that the combination of “intermixing layer” and “implanted layer” formed by the high-energy ion bombardment was the key to enhancing the adhesion between Ag films and Mo substrate effectively.

## 1. Introduction

Silver films are widely used in electrode, interconnection, decorative coating, reflector mirror, and transparent conducting films, etc., because of their excellent optical, thermal and electrical properties. Among the various applications, interconnection, which shows high conductivity (about 6.3 × 10^7^ m^−1^Ω^−1^) and also nice weldability, is used for interconnected materials to provide a current path between single cells for solar cell arrays in low earth orbit (LEO) spacecraft [1,2,3,4,5]. However, in the presence of atomic oxygen (AO, the AO reaction rate of Ag is about 10.5 × 10^−24^ cm^3^ atom^−1^ [4]), Ag is easily corroded and oxidized by AO, leading to serious damage: electrical conductivity and mechanical strength losses, as well as flaking off of the material. With the development of interconnected materials, silver-coated molybdenum interconnectors have replaced the traditional Ag interconnectors [3,4,5,6,7,8,9]. Generally, the adhesive strength of the Ag/Mo interface should be larger than 10 MPa to sustain the machining stress in the parallel gap resistance welding process and later use. Nevertheless, in immiscible Ag-Mo systems, the enthalpy of intermixing is extremely large (about + 57 KJmol^−1^). Additionally, the coefficient of thermal expansion (CTE) of Ag and Mo is a serious mismatch (the CTE of Ag and Mo is 19.5 × 10^−6^ K^−1^ and 5.2 × 10^−6^ K^−1^ respectively). Therefore, it is difficult to form the metallurgical bonding or compound between the Ag films and the Mo substrate.

Films/substrate adhesive strength is mainly influenced by the surface state (including surface energy, roughness and stress, etc.) of the substrate, the bonding mode (mechanical interlocking, metallurgical bonding or compound) between films and substrates, and the interior stress of films, etc. [10,11,12,13]. As a result, coarsening the surface of the substrate, forming compound in the interface and depositing an adhesive transition layer on the surface of the substrate are some common methods of improving the adhesion of the films on the substrate used in recent studies [12,13,14,15,16,17]. Additionally, ion beam-assisted technology, which is based on ionized particle bombardment, has been particularly successful in phase formation, heteroepitaxy and stress engineering, as well as for adhesion properties [18,19,20,21,22,23]. Typically, in conventional ion beam-assisted technologies for fabricating thin films, ions with energy between 1 keV and 10 keV are utilized to influence the nucleation, crystallinity, morphology and defect concentration of thin films [24,25,26]. Only a few studies [27] have been reported that investigate the modification of the interface at different energies of ion bombardment.

In this work, the ion beam-assisted deposition (IBAD) method was used to improve the adhesion of silver films to a Mo substrate, where the assisting ion energy was higher than 10 keV. It was demonstrated that this method could be very useful for improving the film/substrate interfacial adhesion. In IBAD, high-energy argon ions were used to bombard the surface of the growing thin films. Since the deposition of the films and the surface sputtering of the substrate material are carried out at the same time in the IBAD process, atomic intermixing between the film and the substrate can occur [28]. Simultaneously, under high-energy ion bombardment, some film atoms can be implanted into the surface of the substrate, as has been reported previously [29]. Considering that the adhesion between film and substrate is strongly influenced by their interfacial bonding state, correlation of the interfacial structure evolution to adhesion change of the Ag films/Mo substrate is investigated for optimization of deposition conditions.

In addition, the correct characterization of the adhesive strength is of major importance for work focusing on the optimization of the interface between the substrate and the coating; therefore, the peel or pull-off test [14,30,31,32] is commonly used. However, the results are usually not accurate enough, due to the uncertainty regarding the area of the pull-off films. In order to improve measurement accuracy of the test, a certain region of the Mo substrate was masked, and the Ag films were only deposited on the exposed zone. As a result, the area of the pulled-off films of the different samples could be accurately determined.

## 2. Materials and Methods

### 2.1. Sample Preparation

Commercially available Mo sheets (Xi’an Gemei Metal Material Co., Ltd., Xi’an, China) were selected as the substrate material and cut into square-shaped samples of approximately 10 mm × 10 mm × 1 mm, with one side surface mirror polished. The composition of the Mo sheets is listed in Table 1. These Mo specimens were ultrasonically cleaned with acetone, ethanol and deionized water in sequence before being dried. Prior to the film deposition, all Mo substrate specimens were sputtering-cleaned by 0.6 keV Ar^+^ with the beam-current density of 0.76 mAcm^−2^ for about 10 min.

The deposition of polycrystalline Ag films was performed on a homemade deposition system that has been described elsewhere [33]. In this study, the Mo substrate was firstly treated via IBAD method with different ion energies. Subsequently, the Ag films were deposited on these treated Mo substrates by MD method.

During the IBAD process, the sputtering ion source was used for sputtering an Ag target (purity >99.99%) with Ar^+^ ions energy of 2.5 keV and a beam-current density of 3.06 mAcm^−2^, while an assisting ion beam normal to the substrate surface was used for bombarding the films. The assisting ion beam energy was set to 10 keV, 15 keV, 20 keV, 25 keV, 30 keV and 35 keV, respectively, and the beam-current density was kept constant at 0.025 mAcm^−2^. The deposition lasted for 40 min under Ar atmosphere with a constant pressure of 1.7 × 10^−2^ Pa.

For magnetron sputtering deposition (MD), the sputtering devices were also installed in the same chamber. The distance between sputtering target and substrates was 55 mm. The working pressure was set to 1 Pa, with argon flow kept at 60 sccm. The DC power supplied to the Ag target (purity >99.99%, 2-inch diameter) was maintained at 120 W. The deposition time was 30 min.

For simplicity, based on the different energies of the assisting ion beam, these specimens were named as “*x*keV IBAD/MD-Ag”(*x* denotes the energy value of the assisting ion beam, e.g., 10 keV IBAD/MD-Ag). As a comparative test, specimens carried out under the same experimental conditions but without IBAD process were named as “MD-Ag”. During the deposition, the substrate was kept rotating with a speed of 4 rpm in order to achieve a uniform film thickness. The total thickness of the silver films was about 4.5 μm for the MD-Ag and about 5 μm for the IBAD/MD-Ag.

### 2.2. Evaluation

**Figure 1:**
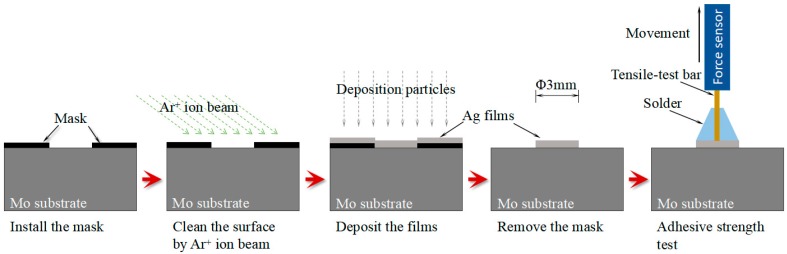
Schematic representation of the specimen preparation for pull-off test.

The adhesion was evaluated by using a tensile pull-off tester (HZ-1007C, Shanghai Hengzhun instrument technology Co., Ltd., Shanghai, China). To achieve the same pulled-off film area of all specimens, a mask was used during film deposition. A simplified sketch of the specimens prepared for the pull-off test is shown in Figure 1. Prior to deposition, a mask with a hole of diameter 3 mm was installed on the surface of the Mo substrate. After the deposition of the thin films, the mask was removed. Subsequently, deposition of 3 mm diameter films is carried out on the substrate via different processes. The adhesive strength P between films and substrate is expressed as

P = F/A(1)

where A and F represent the area of the pulled-off Ag thin films and the maximum force when the Ag thin films are pulled off from Mo substrate, respectively. The area of the pulled-off region was calculated to be about 7.1 mm^2^, with a diameter of 3 mm. The test was repeated at least five times for the specimens prepared by the same technology.

The crystal orientation of the Ag thin films was examined by X-ray diffractometer (XRD, D8 Advance, Bruker, Billerica, MA, USA) with Cu Kα radiation in the 2θ range of 25°–90°. A scanning electron microscope (SEM, S4800, Hitachi, Tokyo, Japan) was employed to investigate the cross-section morphology of the MD-Ag and 30 keV IBAD/MD-Ag. A scanning electron microscope (SEM, EVO-MA10, ZEISS, Oberkochen, Germany) was employed to investigate the surface morphology of the pulled-off region for the MD-Ag, 30 keV IBAD/MD-Ag and 35 keV IBAD/MD-Ag after the pull-off test. X-ray photoelectron spectroscopy (XPS, K-Alpha 1063, Thermo Fisher, Waltham, MA, USA) was used to measure depth profile of the samples and phase compositions on the pulled-off region of the samples after pull-off test.

## 3. Results and Discussion

**Figure 2:**
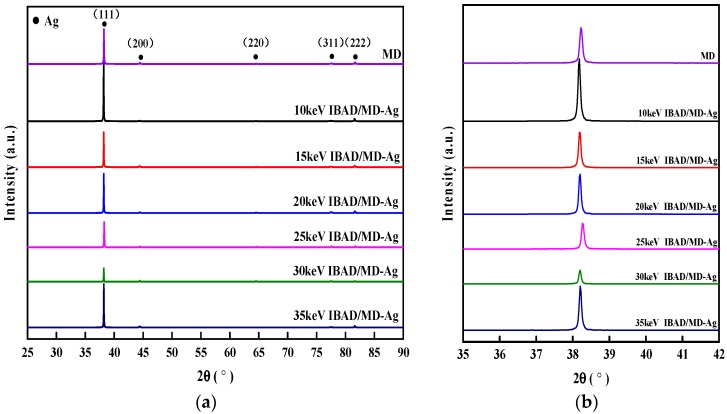
(**a**) XRD spectra of silver films prepared by different process methods; and (**b**) the enlarged diffraction peak (111) pattern from 35 to 42 degrees.

Polycrystalline thin films, grown by any method, would display a certain degree of crystallographic texture [34,35,36,37,38]. A lot of research regarding Ag films prepared by the conventional magnetron sputtering confirms that the films often exhibit a preferred (111) orientation, while those bombarded by the ion beam would transform to (100) or (110) [36,37,38]. Some reports illustrate that the mechanical and physical properties of thin films are also affected by their texture [34,39]. As a consequence, in order to avoid the effect of crystal orientation, micro-structure and grain size on adhesive strength, these affecting factors should be as similar as possible. The XRD spectra of the silver-coated Molybdenum under the different deposition conditions are shown in Figure 2. Comparedto the JCPDS PDF No. 04-0783^#^, the detailed analysis demonstrates that typical peaks of crystal planes of silver are discerned. All the films exhibit a very strong (111) preferred orientation due to the lowest surface energy of the plane. Only a few weak diffraction peaks of Ag (200), (220), (311) or (222) can be found from the results of some specimens. As can be seen in Figure 2b, the full width at half maximum (FWHM) values of the Ag films deposited by MD or IBAD/MD are also similar. This indicates that the average grain size of the Ag films estimated roughly by Debye-Scherrer equation is very similar for all specimens. Therefore, the above-mentioned results illustrate that the orientation and grain sizes of the Ag films are relatively consistent when prepared under the different deposition conditions in this work.

**Figure 3:**
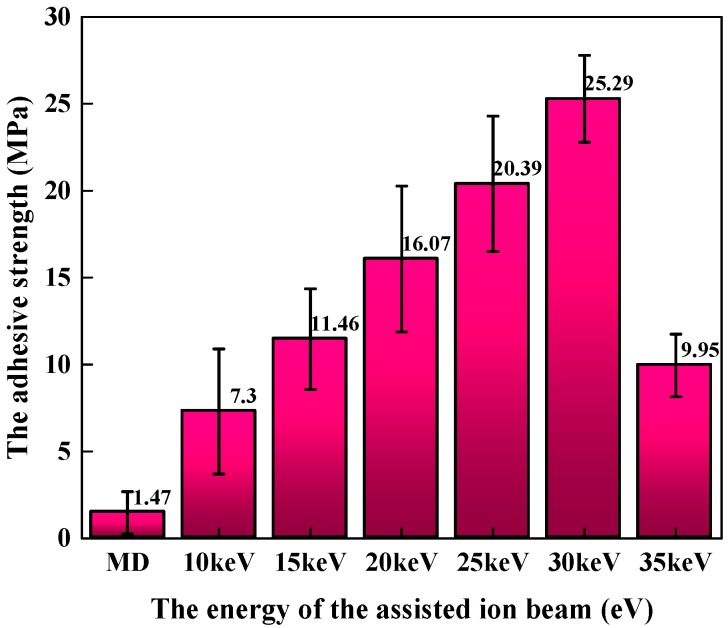
Relationships between the adhesion strength and the assisting ion beam energy.

As mentioned above, the force used to separate the films from the substrate is mainly determined by the interaction of several factors: surface structure and stress states of the substrate, bonding mode between the films and the substrates, structure and properties of the films, and the process used to prepare the films, etc. Once one of these parameters is changed, the pull-off strength can be used to quantify the influence of this parameter on the adhesion strength. Figure 3 shows the influence of the different energies of assisted ion beam on the adhesive strength of silver-coated Mo substrates. As expected, the adhesive strength is greatly enhanced by the bombardment of the high-energy ion. Even for the 10 keV IBAD/MD-Ag, its adhesive strength is approximately 5 times higher than that of the MD-Ag. It also can be seen that the adhesive strength first increases throughout the energy range from 10 keV to 30 keV, and then decreases at 35 keV with the increasing the energy of the assisting ion beam. The maximum value of the adhesive strength reaches 25.29 MPa when the energy of the assisted ion beam is 30 keV.

**Figure 4:**
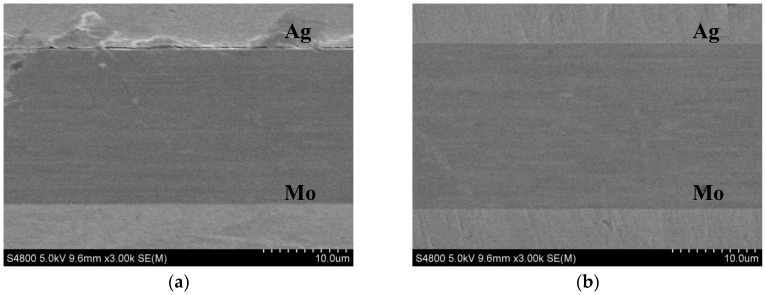
SEM micro-graphs of the cross sections of (**a**) MD-Ag and (**b**) 30 keV IBAD/MD-Ag.

As shown in Figure 4, the cross-section morphology of the MD-Ag and the 30 keV IBAD/MD-Ag respectively was determined by scanning electron microscopy (SEM). From Figure 4a, a clear crack can be seen at the film/substrate interface, and it can be inferred that the Ag film had flaked off from the substrate during the polishing. This further confirms that the adhesive property of the MD-Ag is extremely poor. However, no cracks or pores are found in the interface of the film/substrate from Figure 4b. It implies that the adhesive strength is effectively enhanced by IBAD.

**Figure 5:**
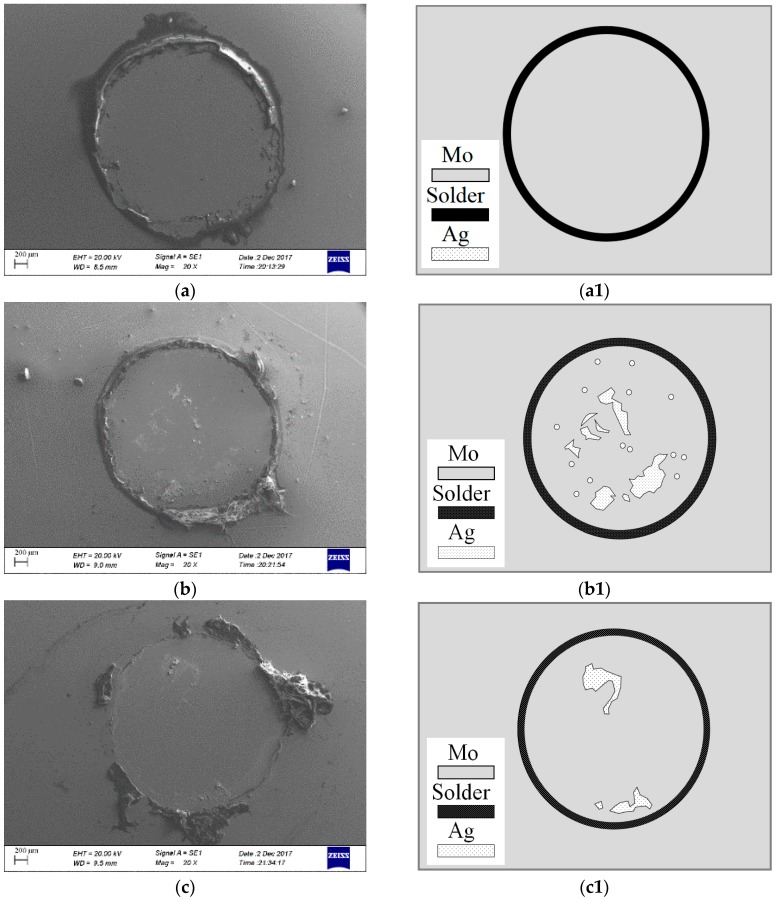
Surface morphology of pulled-off region for (**a**) the MD-Ag, (**b**) 30 keV IBAD/MD-Ag, and (**c**) 35 keV IBAD/MD-Ag; and their sketches after pull-off test.

To further analyze the failure modes of the films, the surface morphology of the adhesion region after the pull-off test were studied by SEM. For ease of observation and comparison, the SEM images of the MD-Ag, 30 keV IBAD/MD-Ag and 35 keV IBAD/MD-Ag after the pull-off test and their sketches are shown in Figure 5. The results show that the interface failure mechanism is found in the adhesion region without solder failure for all specimens. The interface failure is expected as a result of both the lower interfacial adhesion and high tensile strength of the coating itself. As seen in Figure 5a(a1), the pulled-off region is extremely smooth and clear, and almost no remnants are found. It can be inferred that the film/substrate adhesion mainly relies on Van der Waals forces for the MD-Ag. In contrast, the coating delamination and a certain amount of residual Ag films can be found in Figure 5b(b1),c(c1). This indicates that the film/substrate adhesive strength in some adhesion regions is higher than the tensile strength of the coating itself in both 30 keV and 35 keV IBAD/MD-Ag. Nevertheless, the residual Ag films of the 30 keV IBAD/MD-Ag show much more remnants than those of the 35 keV IBAD/MD-Ag, which could explain why the adhesive strength decreased sharply when the energy of the assisting ion beam rises to 35 keV. Combining the above analysis, it can be inferred that some Ag particles were implanted into the surface of Mo substrate under the bombarding of high-energy argon ion, and a similar “piling effect” was carried out, which could strongly improve the mechanical interlocking between the films and the substrate. However, with further increase in the bombardment energy, more Ag particles were implanted into the deeper position, while the Ag particle concentration in the Mo substrate surface decreased. This is consistent with some previous reports [40,41]. Therefore, for the immiscible feature of Ag-Mo system, the inter-atomic mechanical interlocking between the films and the substrate would been weaken due to the reduction of the Ag concentration in the surface of the substrate, causing the adhesive strength decrease.

**Figure 6:**
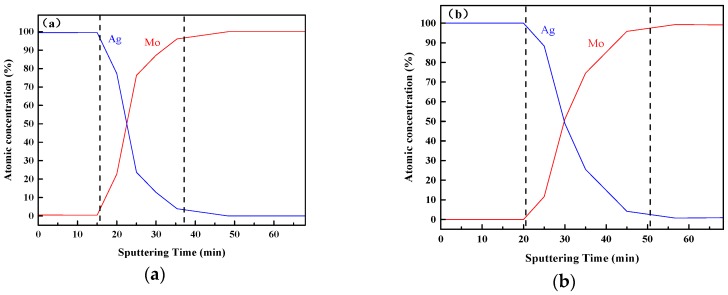
Depth profiles of (**a**) MD-Ag and (**b**) 30 keV IBAD/MD-Ag.

As shown in Figure 6, the depth profile of 30 keV IBAD/MD-Ag and the MD-Ag were analyzed by X-ray photoelectron spectroscopy (XPS), respectively. In order to shorten the sputtering time during the XPS test, the deposition time was set to 3 min for MD and the time for IBAD was kept constant at 40 min. It is clearly found that the gradient distribution of Ag atom along the depth is formed in both the IBAD/MD-Ag and the MD-Ag. Nevertheless, in contrast to the IBAD/MD-Ag, a faster decrease of silver atomic percentage is exhibited in the MD-Ag.

The above-mentioned results show that mutual diffusion of elements can occur between the films and the substrate for both the IBAD/MD-Ag and the MD-Ag. This is because atom deposition of the film material, as well as surface sputtering of the substrate material, can be carried out during the initial deposition with high-energy ion bombardment [42]. Even for the MD-Ag, the same phenomenon is found, may be due to the plasma effect during the sputtering deposition, and this result is consistent with the report of Ran [43]. Considering the deposition rate of IBAD or MD and sputtering rate of the XPS analysis, the width of the mutual diffusion region of the MD-Ag and the IBAD/MD-Ag can be estimated at about 150 nm and 300 nm respectively.

**Figure 7:**
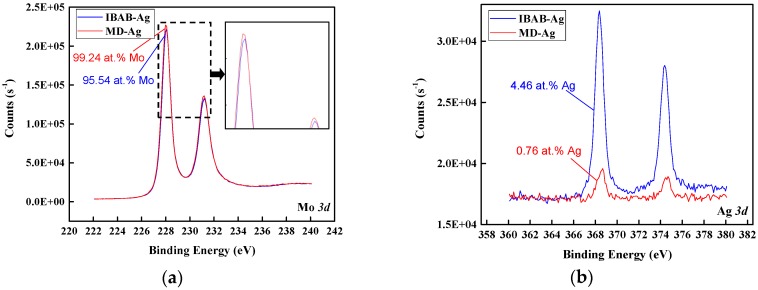
(**a**) Mo3d and (**b**) Ag 3d XPS spectra on the pulled-off region of the 30 keV IBAD-Ag and the MD-Ag after the pull-off test.

In addition, the content of Ag and Mo in the adhesive region surface after the pull-off test was also studied using XPS, and the quantitative analysis (only Ag and Mo elements were retained with normalization processing)of the 30 keV IBAD/MD-Ag and the MD-Ag are shown in Figure 7. It can be seen that the content of Ag and Mo for the 30 keV IBAD/MD-Ag is 4.46 at.% and 95.54 at.%, respectively. However, for the MD-Ag, the content is 0.76 at.% and 99.24 at.%, respectively. These results indicate that the content of Ag in the 30 keV IBAD/MD-Ag is obviously higher than that of the MD-Ag, and this result is also consistent with SEM. Combining this with the results of the XPS depth profiles, it can be further inferred that the implantation effect is quite notable under the bombardment of the high-energy ion [44].

**Figure 8:**
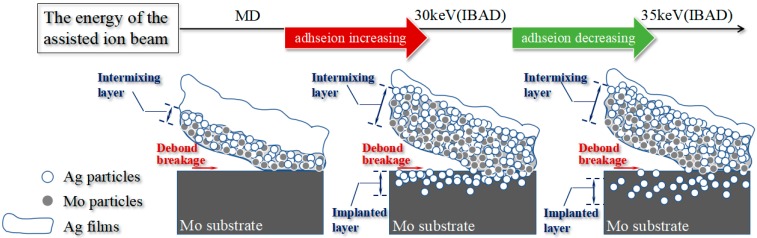
Schematic of the interface morphology evolutions and associated adhesion mechanism of Ag thin films on Mo substrate.

Based on the adhesive strength by pull-off test and the analyses using SEM and XPS, the adhesion mechanism of the Ag films to the Mo substrate has been verified and is illustrated in Figure 8. The adhesive strength is mainly influenced by two main factors: the structure of intermixing layer and the distribution of Ag in the implanted layer. The maximum adhesive strength is obtained at the optimal assisting ion beam energy of 30 keV, which is attributed to the appropriate thickness of the intermixing and the implanted layers. Moreover, the presence of a sufficient amount of Ag particles in the surface of the implanted layer also contributes to the great adhesive strength, as confirmed by the SEM and XPS analyses. In the MD-Ag, the intermixing layer is also present, but is relatively thin, and few Ag particles are implanted into the surface of the Mo substrate. Therefore, the film/substrate adhesion is dominated only by Van der Waals forces. However, in the IBAD/MD-Ag, a wide atomic intermixing layer can be carried out at the interface between the films and substrate, and some Ag particles are implanted into the surface of the substrate at the same time, which strongly improves the mechanical interlocking between the films and the substrate. Moreover, with the further increase of the assisted ion beam energy, the Ag particles may be implanted into a deeper position in the substrate, while the Ag concentration at the surface of the substrate is reduced simultaneously. Therefore, the inter-atomic mechanical interlocking between the films and the substrate will be weakened due to the reduction of the Ag concentration in the surface of the substrate, which decreases the adhesive strength.

## 4. Conclusions

In this paper, an enhancement mechanism for the adhesive strength of the Ag films on the Mo substrate was analyzed and discussed. The pull-off test was used to quantitatively measure the adhesive strength under the different preparation process. The SEM and XPS analyses demonstrated that the adhesive strength was critically influenced by the structure of intermixing layer and the Ag particles distribution of the implanted layer at the interface. The results are as follows:

- Because of its implanted and atom-intermixed effect, IBAD process enhances the adhesive strength between Ag films and Mo substrate effectively.
- With the increasing of energy of the assisted ion beam, the adhesive strength first increased and then decreased due to the concentration change of the Ag particles in the implanted surface layer.
- The maximum value of the adhesive strength of Ag films on Mo substrate reaches 25.29 MPa.

## Figures and Tables

**Table 1:** Chemical composition of Mo sheets (wt.%).

Element	Al	Ca	Fe	Mg	Ni	Si	C	N	O	Mo
Content	0.002	0.002	0.01	0.002	0.005	0.01	0.01	0.003	0.008	Bal.
